# A Comparison between the Sixth and Seventh Editions of the UICC/AJCC Staging System for Nasopharyngeal Carcinoma in a Chinese Cohort

**DOI:** 10.1371/journal.pone.0116261

**Published:** 2014-12-23

**Authors:** Jing Li, Xiong Zou, Yun-Long Wu, Jing-Cui Guo, Jing-Ping Yun, Miao Xu, Qi-Sheng Feng, Li-Zhen Chen, Jin-Xin Bei, Yi-Xin Zeng, Ming-Yuan Chen

**Affiliations:** 1 Collaborative Innovation Center for Cancer Medicine, State Key Laboratory of Oncology in South China, Sun Yat-sen University Cancer Center, Guangzhou 510060, P. R. China; 2 Department of Experimental Research, Sun Yat-sen University Cancer Center, No. 651, Dongfeng East Road, Guangzhou 510060, P. R. China; 3 Department of Nasopharyngeal Carcinoma, Sun Yat-sen University Cancer Center, No. 651, Dongfeng East Road, Guangzhou 510060, P. R. China; 4 Department of Pathology, Sun Yat-sen University Cancer Center, No. 651, Dongfeng East Road, Guangzhou 510060, P. R. China; 5 Department of Oncology, the First Affiliated Hospital of Zhengzhou University, Zhengzhou 450052, China; University of Nebraska Medical Center, United States of America

## Abstract

**Background:**

The International Union Against Cancer/American Joint Committee on Cancer (UICC/AJCC) TNM staging system of nasopharyngeal carcinoma (NPC) is the most important system for survival prediction. The TNM 7th edition UICC/AJCC TNM staging system for NPC was adopted in January 2009, and is now internationally recommended. In comparison with the TNM 6th edition, there were several revisions in the new edition staging system. This study aims to evaluate the prognostic value of the TNM 7th edition for NPC patients in comparison with the TNM 6th edition.

**Method:**

Clinical data of 2,629 NPC patients from the Sun Yat-sen University Cancer Center between January 2006 and December 2010 were retrospectively collected and all the patients were restaged according to the criteria of the TNM 6th edition and TNM 7th edition UICC/AJCC staging manual. Univariate and multivariate COX proportional hazards analyses were applied to evaluate the prognostic values between adjacent stage categories of the TNM 6th edition and TNM 7th edition.

**Results:**

In comparison with the TNM 6th edition, a significant alteration of the distribution of N categories was observed when the TNM 7th edition was applied (χ^2^ = 20.589, P<0.001), with 119 (119/670, 17.8%) patients up-staging from N0 to N1. With regard to T and overall stage, 37 (37/561, 6.6%) patients were down-staged from T2a with the TNM 6th edition to T1 with the TNM 7th edition, and finally two patients were up-staged to overall stage II (2/118, 1.7%). Moreover, the survival curves were significantly segregated (P<0.05) between T1 and T2 as well as N1 and N2 with the TNM 7th edition.

**Conclusions:**

The TNM 7th edition led to a significant alteration in the distribution of N categories and it is superior to the TNM 6th edition in predicting the frequency of overall survival and distant metastasis-free survival.

## Introduction

Nasopharyngeal carcinoma (NPC) is a non-lymphomatous and squamous-cell carcinoma, which is commonly associated with Epstein-Barr virus infection [1]. The annual incidence of NPC reaches about 25 per 100,000 individuals among the Cantonese people that inhabit Guangdong province in Southern China, which is 25-fold higher than that in the Western world [1], [2]. During the past decades, the overall 5-year survival rate has improved substantially from 60% to 80%. However, 20–30% patients finally progress with distant metastasis and/or loco-regional recurrence [3], [4]. Therefore, better treatment approaches and rational planning are urgently required for NPC patients.

As outlined by the UICC/AJCC, the TNM staging system is the most important prognosticator for survival prediction and contributes largely to the treatment planning of NPC patients [5], [6]. To date, there have been four editions of TNM staging systems for NPC that have been updated in association with the development of imaging and treatment technologies. The TNM 6th edition has been commonly used since its release in 2002 [7]; however, there are still some controversies: firstly, accumulative studies revealed that nasal and/or oropharyngeal invasion (T2a by TNM 6th edition) without parapharyngeal extension had a similar prognosis compared to T1; secondly, retropharyngeal lymph node (RLN) involvement, regardless of laterality, had an inferior prognosis compared to node-negative disease[8], [9], [10]; thirdly, masticator space invasiveness including the medial and lateral pterygoid muscles had a similar prognosis to T4 [11], [12]. In 2009, the TNM 7th edition UICC/AJCC staging system for NPC was released [13] and revised the corresponding criteria based on the previous findings except for the definition of the masticator space. In fact, there was no instruction about the definition of masticator space in the TNM 6th edition and TNM 7th edition AJCC cancer staging handbook [14], [15]. Therefore, there was no obvious change regarding T4. Here, in order to evaluate the prognostic value of the TNM 7th edition, we compared the prognostic performance of the TNM 7th edition with the TNM 6th edition, in a large retrospective cohort of 2,629 NPC patients.

## Materials and Method

### Patient characteristics

We retrospectively reviewed the medical records of 2,671 consecutive patients with biopsy-proven hospitalization at Sun Yat-sen University Cancer Center (SYSUCC) between January 2006 and December 2010. One patient without radiation therapy and 41 patients without a complete medical history of clinical and follow-up data were excluded from the study. Finally, 2,629 patients remained for further analyses (Table 1 and S1 Table). This study was approved by the Institutional Review Board of the Sun Yat-sen University Cancer Center. All patients signed an informed consent form prior to participating in the study. All 2,629 patients had undergone fiberoptic endoscopic biopsy of the nasopharynx and contrast-enhanced CT/MRI of the nasopharynx and neck during staging work-up. Almost all individuals (2,625/2,629, 99.8%) received magnetic resonance imaging (MRI) of the head and neck to evaluate the primary tumor and the frequency of LN metastases, and the other four patients received CT. All included patients underwent other pretreatment evaluations, including physical and neurologic examinations, hematology and biochemistry profiling, chest radiography, abdominal ultrasonography and emission CT scan. In addition, a total of 287 (10.9% of 2,629) patients underwent 18F-fluorodeoxyglucose positron emission tomography and computed tomography (PET/CT) examination to help evaluate distant metastatic disease. Cranial nervepalsy was assessed clinically. Two clinicians independently reviewed all of the images based on the MRI/CT diagnosis criteria and re-staged all the patients according to the criteria of the TNM 6th edition and TNM 7th edition for NPC (Table 2). Any disagreements were resolved by consensus.

**Table 1:** Distribution and characteristics of 2,629 patients with nasopharyngeal carcinoma.

Characteristic	TNM 6th edition		TNM 7th edition			
	N	%	N	%	?^2^	P
**Gender**						
Female	673	25.6	673	25.6		
Male	1,956	74.4	1,956	74.4		
**Age (year)**						
Median	50		50			
Range	6–98		6–98			
**Histology**						
WHO I+II	142	5.4	142	5.4		
WHO III	2,487	94.6	2,487	94.6		
**Treatment**						
RT	673	25.6	673	25.6		
CRT	1,956	74.4	1,956	74.4		
**Radiotherapy techniques**						
2D-CRT	1,863	70.9	1,863	70.9		
3D-CRT	85	3.2	85	3.2		
IMRT	681	25.9	681	25.9		
**T-classification**					3.467	0.325
T1	292	11.1	329	12.5		
T2	561	21.3	524	19.9		
T3	1,247	47.5	1,247	47.5		
T4	529	20.1	529	20.1		
**N-classification**					20.589	<0.001
N0	670	25.5	551	21		
N1	728	27.7	847	32.2		
N2	1,042	39.6	1,042	39.6		
N3	189	7.2	189	7.2		
**M-classification**						
M0	2,553	97.1	2,553	97.1		
M1	76	2.9	76	2.9		
**Overall Stage**					0.22	0.999
I	118	4.5	120	4.6		
II	389	14.8	387	14.7		
III	1,400	53.3	1,400	53.2		
IV	722	27.5	722	27.5		

Abbreviations: AJCC  =  American Joint Committee on Cancer; WHO  =  World Health Organization; RT  =  Radiotherapy; CRT  =  Chemoradiotherapy; 2D-CRT  =  conventional 2-dimensional radiotherapy; 3D-CRT  =  3-dimensional conformal radiotherapy; IMRT  =  Intensity-modulated radiation therapy

**Table 2:** Classification criteria of TNM 6th and TNM 7th staging system for nasopharyngeal carcinoma.

	TM6th	TNM7th
**T classification**		
**T1**	nasopharynx	nasopharynx, oropharynx or nasal cavity
**T2**		
**T2a**	oropharynx and/or nasal cavity	pharapharyneal extension
**T2b**	parapharyneal extension	
**T3**	bony structures and/or paranasal sinuses	bony structures and/or paranasal sinuses
**T4**	intracranial extension and/or cranial nerves, infratemporal fossa hypophaynx, orbit or masticatory space^a^	intracranial extension and/or cranial nerves, hypopharynx, orbit or infratemporal fossa/masticatory space^b^
**N classification**		
**N0**	none	none
**N1**	unilateral node(s), ≤6cm in greatest dimension, above the supraclavicular fossa	unilateral cervical and/or unilateral or bilateral retropharyngeal node(s),≤6cm in greatest dimension, above the supraclavicular fossa
**N2**	bilateral node(s),≤6cm in greatest dimension, above the supraclavicular fossa	bilateral cervical node(s),≤6cm in greatest dimension, above the supraclavicular fossa
**N3a**	>6cm, or in supraclavical fossa	>6cm, or in supraclavical fossa
**stage group**		
**I**	T1N0M0	T1N0M0
**II**		T1N1M0, T2N0-1M0
**IIA**	T2aN0M0	
**IIB**	T1-2aN1M0, T2bN0-1M0	
**III**	T1-2bN2M0, T3N0-2M0	T1-2N2M0, T3N0-2M0
**IVA**	T4N0-2M0	T4N0-2M0
**IVB**	any T N3M0	any T N3M0
**IVC**	any T any N 3M1	any T any N 3M1

Abbreviations: UICC = the International Union Against Cancer; AJCC  =  American Joint Committee on Cancer

a Masticator space involvement denotes extension of tumor beyond the anterior surface of the lateral pterygoid muscle or lateral extension beyond the posterolateral wall of the maxillary antrum and the pterygomaxillary fissrue.

b Masticator space primarily consists of the muscles of mastication. Anatomically, the superficial layer of the deep cervical fascia splits to enclose the muscles of mastication to enclose this space. These muscles are the medial and lateral pterygoid, masseter and temporalis.

With regard to non-distant metastatic cancer, it was defined as a primary tumor with or without local regional lymphocyte node metastases, including stage I, II, III, IVa and IVb subgroups. With respect to distant metastasis, it was defined as a tumor spreading from the original (primary) tumor to distant organs or distant lymph nodes. Among a total of 2,629 individuals recruited, 542 patients had a primary tumor without regional lymphatic metastases, 2,011 patients had local regional lymphatic metastases, and the remaining 76 patients had distant metastases.

### Determination of nasal and/or oropharyngeal cavity invasion for the T category

Diagnostic MRI/CT criteria for nasal invasion included nasal structure (turbinates and nasal septum) involvement, and extension to the line of bilateral pterygopalatine fossa. Oropharyngeal invasion was determined as the tumor margin accessing the gap of the first and second cervical intervertebra [16]. Patients with nasal cavity and/or oropharynx involvement were classified as T2a by the TNM 6th edition but T1 by the TNM 7th edition [17].

### Determination of lymph node metastasis for N classification

Metastatic lymphadenopathy was determined by using MRI/CT with any of the following: 1) lateral RLNs with a minimal axial dimension (MID) of 6 mm [18] and any lymph node observed in the median retropharyngeal group, or cervical lymph nodes with a MID of 11 mm in the digastric region, and MID of 10 mm for all other cervical lymph nodes except the retropharyngeal group; 2) lymph nodes of any size with central necrosis or a contrast-enhancing rim; and 3) lymph node cluster, meaning the presence of 3 contiguous and confluent lymph nodes, each of which should have a MID of 8–10 mm [16], [19]. Patients with RLN metastasis but no positive cervical lymph nodes (N0_RLN+_) were staged as a N0 subset in the 6th edition but an N1 subset in the 7th edition. Patients with neither RLN nor cervical lymph node metastasis (N0_RLN-_) remained in the N0 subset in both editions [17].

### Treatment and follow-up

Radiotherapy with or without chemotherapy remained as standard care for NPC patients [20]. For non-distant metastatic NPC, all patients were treated with standard curative radical radiotherapy, including conventional 2-dimensional radiotherapy (2D-CRT) or intensity-modulated radiation therapy (IMRT), as described previously [20]. Briefly, all target volumes were outlined slice by slice in the treatment planning system based on enhanced CT scans. The radiation dose was 60∼72 Gy at the nasopharyngeal region and 50∼66 Gy at the regional lymph nodes. Part of the patients with stage II and all patients with stage III to stage IV disease were commonly combined with sequential chemotherapy with a platinum-based regimen. Salvage treatments (including surgery, brachytherapy, and chemotherapy) were provided in the event of documented disease recurrence or persistence.

For distant metastatic NPC, loco-regional radiotherapy with cisplatin-based systemic chemotherapy was provided to the patients [21]. Local treatment of metastatic sites, including radiation therapy, surgical resection or ablation, or other treatments were provided to the metastatic patients based on the discretion of the attending radiation oncologists.

The patients were followed up every three months in the first three years, and every six months thereafter or until death. The last follow-up date was May 30, 2013 for all available patients. Local recurrence was confirmed by fiberoptic endoscopy, MRI and biopsy. Distant metastases were diagnosed based on clinical symptoms, physical examination, and imaging methods including CT-scan, bone scan, and abdominal sonography or PET-CT.

### Statistical Analysis

The differences in T, N and overall stage distributions between the TNM 6th and TNM 7th editions were compared using chi-square tests. The following endpoints were assessed: overall survival (OS), loco-regional recurrence-free survival (LRRFS), and distant metastasis-free survival (DMFS). The survival rate of OS, LRRFS and DMFS at 3 years and 5 years in percent are supplied in S2 Table.

The event for OS was defined as the duration from diagnosis until the date of death from any causes, or date of the last follow-up. LRRFS was the duration between the date of being diagnosed and the date of having event of loco-regional recurrence or date of the last follow-up. DMFS was defined as the duration from diagnosis until the date of metastasis, or date of the last follow-up. These endpoints were analyzed with a Kaplan-Meier method and a log-rank test. Multivariate analyses with the Cox proportional hazards model were used to test independence, significance, and hazard discrimination. Additionally, survival curves were plotted using the Cox multivariate model in addition to the Kaplan-Meier method. A two-tailed P value <0.05 was considered statistically significant. Above analyses were carried out using SPSS software (version 16.0, SPSS Inc., Chicago, IL, USA).

## Results

### Distributions and characteristics of NPC patients according to the TNM 6th edition and TNM 7th edition

The patients' distributions and characteristics are summarized in Table 1. The median age of the 2,629 patients was 50 years old (6–81 years old) and 1,956 (74.4%) were male. The median follow-up was 54.40 months (range; 1.23–87.20 months). The patients were treated with 2D-CRT (70.9% or 1,863 individuals), 3D-CRT (3.2% or 85 individuals), or IMRT (25.9% or 681 individuals) radiation therapies. The majority of the patients (1,956/2,629, 74.4%) were treated with combination chemoradiation therapy. Also, 622 (23.7%) patients were given induction chemotherapy (IC); 703 (26.7%) patients were given concurrent chemotherapy (CC); 16 (0.6%) patients were treated with adjuvant therapies. Furthermore, 545 (20.7%) patients were given IC and CC treatments and 28 (1.1%) patients were treated with CC and adjuvant chemotherapy. By comparing the distributions of the TNM 6th edition and TNM 7th edition, 37 (37/561, 6.6%) patients were down-staged from T2a with the TNM 6th edition to T1 with TNM 7th edition and 119 (119/670, 17.8%) patients were up-staged from N0 with the TNM 6th edition to N1 with the TNM 7th edition. Eventually, because of the presence of positive lymph nodes (N1-3) and T2-4 subsets, 2 patients were up-staged from stage I to II (2/118, 1.7%). Significant differences in the distribution between the TNM 6th edition and TNM 7th edition were found in N classification (χ^2^ = 20.589, p<0.001) but not in T classification (χ^2^ = 3.476, p = 0.325) or overall stage (χ^2^ = 0.220, p = 0.999; Table1).

### Survival analyses of T categories according to the TNM 6th edition and TNM 7th edition

**Figure 1:**
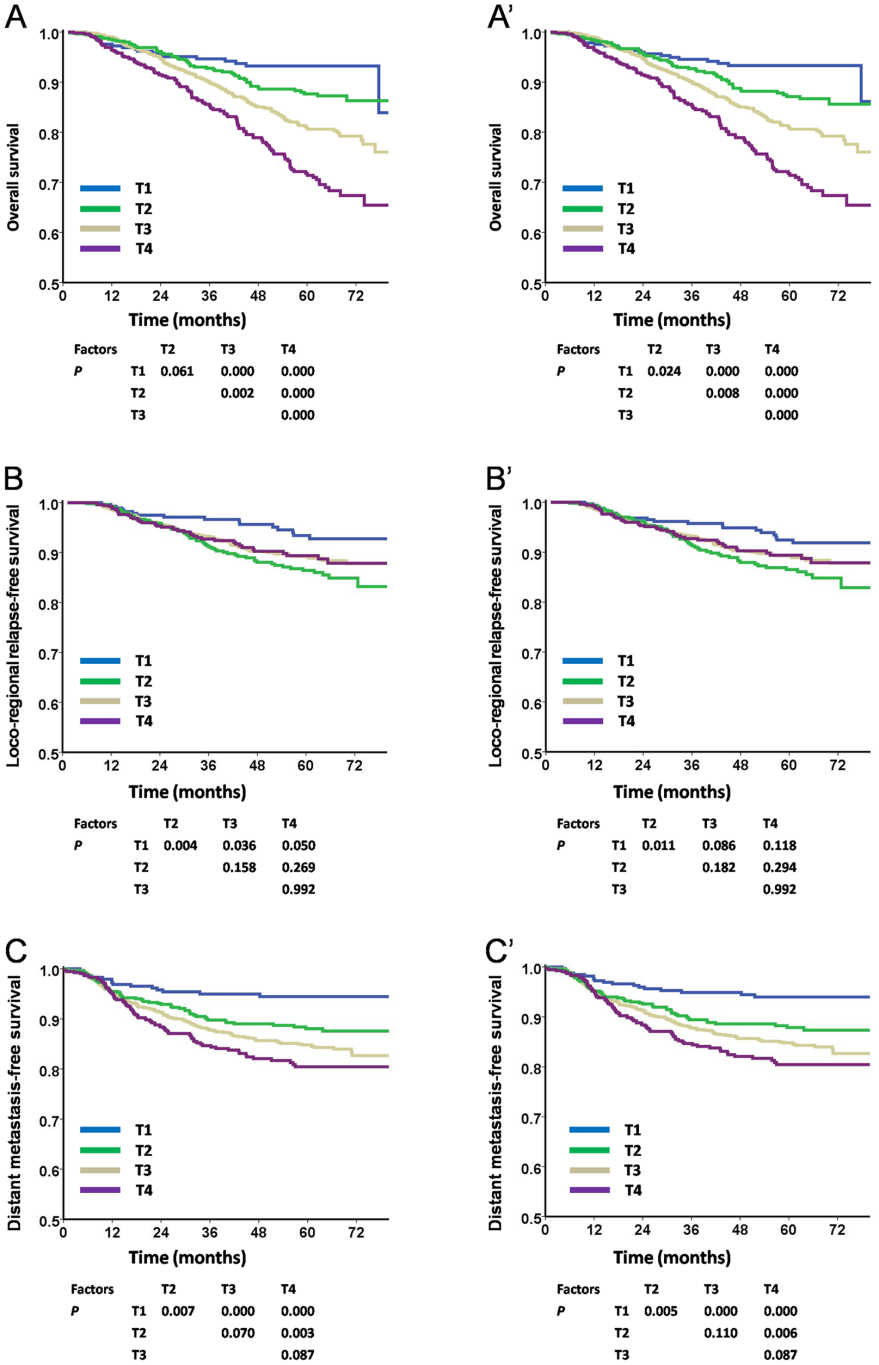
Survival analyses of T categories using the TNM 6th and the TNM 7th editions staging systems. **Panels** A, B, and C were staged according to the TNM 6th edition, while panels A′, B′, and C′ were staged using the TNM 7th edition. In OS analysis, there were significant differences for each stage of T categories except for T1 vs. T2 in the TNM 6th edition (**Panel 1**A), which became significant in the TNM 7th edition (**Panel 1**A′). With regard to LRRFS, no significant differences were observed for each stage of T2-T4 subsets in both editions (**Panels 1**B and1B′). With regard to DMFS, both TNM 6th and TNM 7th editions showed distinct differences in survival among each stages of T categories except for T2 vs. T3 and T3 vs. T4 (**Panels 1**C **and 1C**′). UICC/AJCC: International Union Against Cancer stage system/American Joint Committee on Cancer; OS, overall survival; DMFS, distant metastasis-free survival; LRRFS, loco-regional recurrence-free survival.

For OS, significant separations between adjacent stage categories were observed with the TNM 7th edition. However, the differences between T1 and T2 categories were not significant (P = 0.061) with the TNM 6th edition (Fig. 1A, and Fig. 1A′). With regard to LRRFS, there were nonsignificant survival curve segregations between adjacent stage categories of T2-T4 by both editions of the staging system (Figs. 1B and1B′). For DMFS, survival curves could be significantly segregated among each category of T classification (P<0.05) with both the TNM 6th edition and TNM 7th edition except for T2 vs. T3 and T3 vs. T4 (P>0.05; Figs. 1C and 1C′).

**Figure 2:**
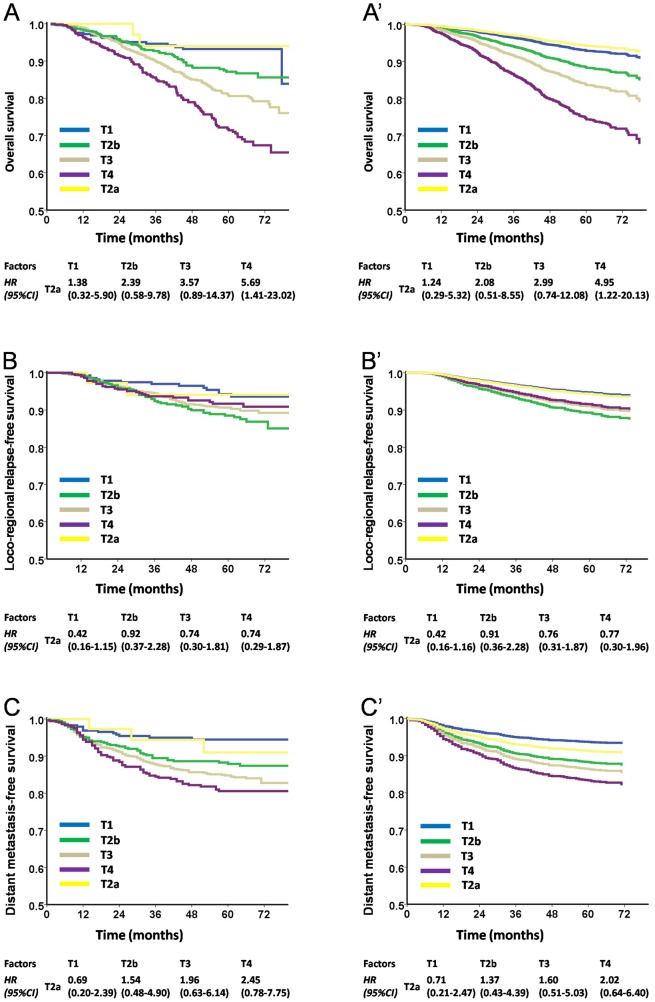
Survival analyses of the HRs of the T2a subset compared with other subsets using the TNM 6th edition staging system. **Panels** A, B, and C were staged according to the TNM 6th edition staging system, while panels A′, B′, and C′ were plotted as estimated from the Cox multivariate model with adjustments for sex, age, histology, treatment modality, and radiotherapy techniques. In both statistical model, the hazard ratios (HRs) of T2a were similar to T1 in OS (**Panels** A and A′), LRRFS (**Panels** 2B and 2B′) and DMFS (**Panels** C and C′) but were significantly different from the T2b subset (parapharyngeal space invasion). HR: hazard ratio.

Further, we classified patients with nasal and/or oropharyngeal cavity invasion (T2a by the TNM 6th edition) as a separate subgroup. Our results showed that the hazard ratios (HRs) of T2a were similar to T1 (nasopharyngx invasion) in OS (before adjusted: HR = 1.38; after adjusted: HR = 1.24; Figs. 2A and 2A′), LRRFS (before adjusted: HR = 0.9; after adjusted: HR = 0.71; Figs. 2B and 2B′) and DMFS (before adjusted: HR = 0.69; after adjusted: HR = 0.71; Figs. 2C and 2C′), but differed significantly from T2b (parapharyngeal space invasion).

### Survival analyses of N categories according to the TNM 6th edition and TNM 7th edition

**Figure 3:**
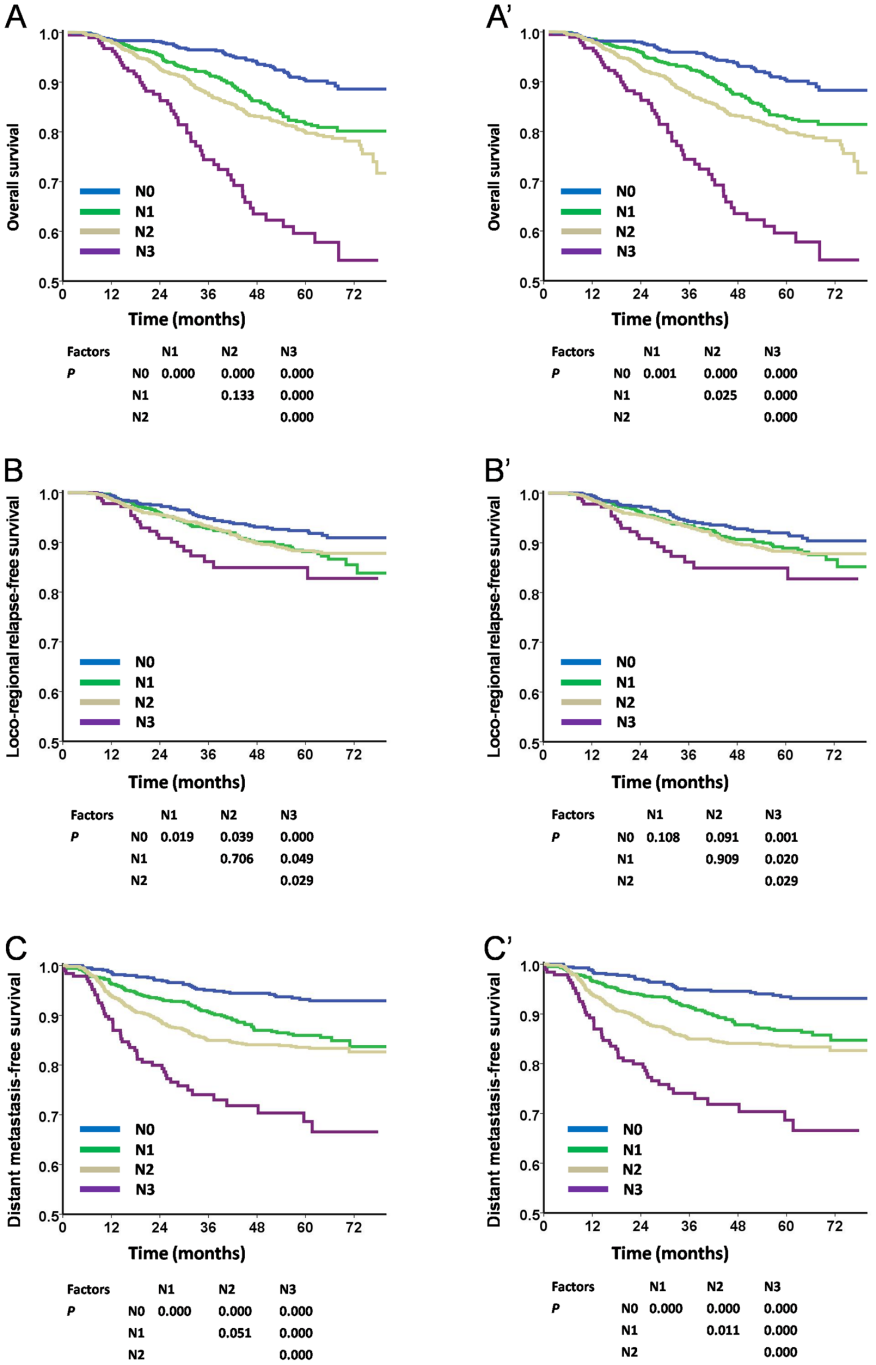
Survival analyses of N categories using the TNM 6th and the TNM 7th editions staging systems. **Panels** A, B, and C were staged according to the TNM 6th edition staging system, while panels A′, B′, and C′ were followed according to the TNM 7th edition. There were significant differences among each stage of N categories except for N1 vs. N2 in either OS or DMFS (**Panels** 3A and 3C) in the TNM 6th edition, while such differences became significant in the TNM 7th edition (**Panels** 3A′ and 3C′). However, neither the TNM 6th nor TNM 7th edition could distinguish the LRFFS between N1 and N2 (**Panels** 3B and 3B′).

Significant survival curve separations were obtained between N1 and N2 with the TNM 7th edition in OS (P = 0.025) and DMFS (P = 0.011; Figs. 3A′ and 3C′). However, these differences were not significant with the TNM 6th edition. With respect to LRRFS, there was a non-significant survival difference between N1 and N2 according to the criteria of the TNM 6th edition and TNM 7th edition (Figs. 3B and 3B′).

**Figure 4:**
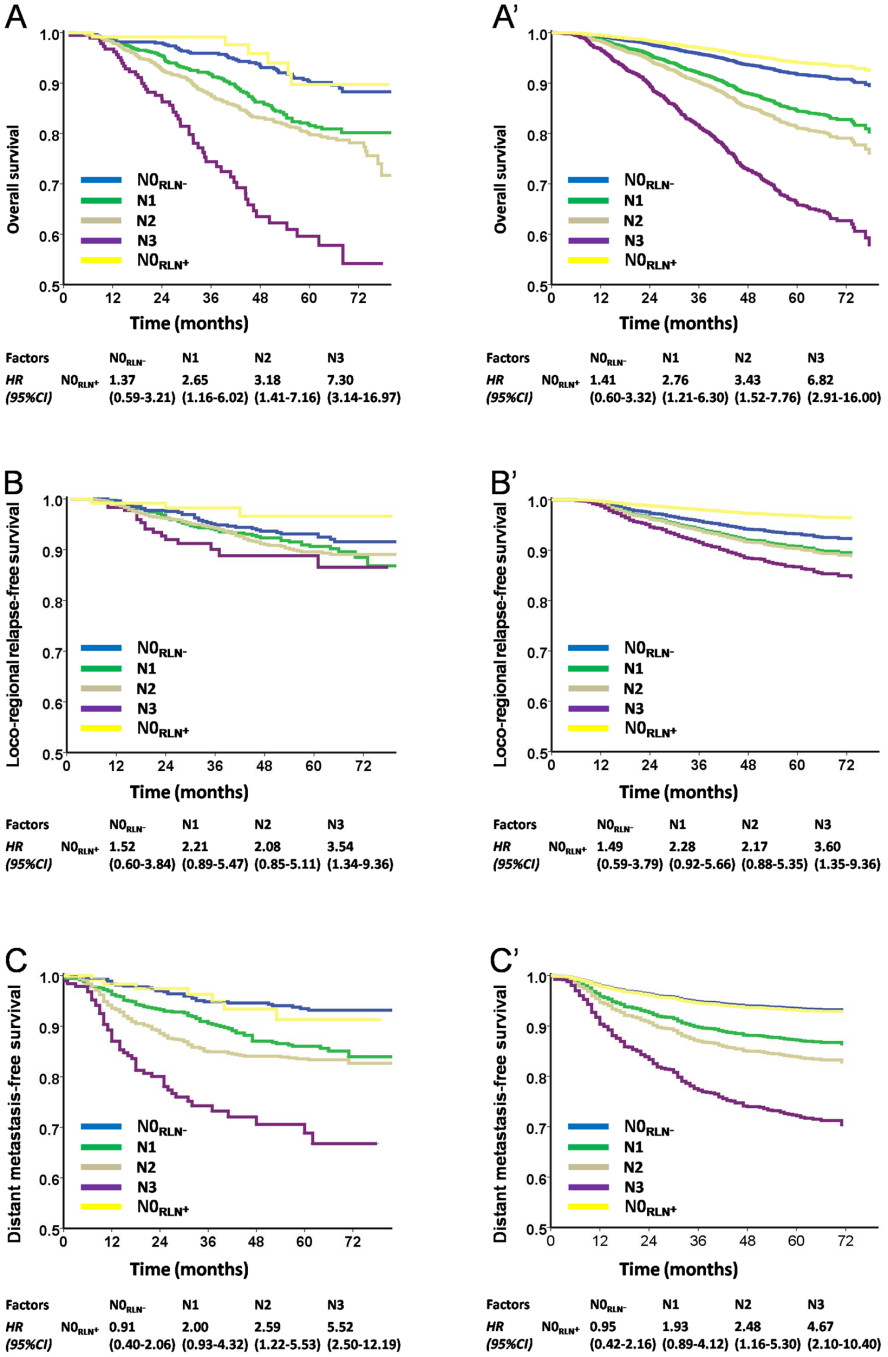
Survival analyses of the HRs of N0_RLN+_ subset comparing with other subsets using the TNM 6th staging system. **Panels** A, B, and C were staged as the TNM 6th edition staging system, while panels A′, B′, and C′ were plotted as estimated from the Cox multivariate model with adjustments for sex, age, histology, treatment modality, and radiotherapy techniques. Patients with N0_RLN**+**_ were divided into a subgroup alone, the HRs of N0_RLN+_ was similar to N0_RLN-_ in OS (**Panels** A and A′), LRRFS (**Panels** B and B′) and DMFS (**Panels** C and C′).

Further, we classified N0 patients with RLN metastasis (N0_RLN**+**_ by the TNM 6th edition) as a subgroup. The HRs of N0_RLN+_ were found to be similar to N0 without RLN metastasis (N0_RLN-_; before adjusted: OS, HR = 1.37, LRRFS, HR = 1.52 and DMFS, HR = 0.91; after adjusted: OS, HR = 1.41, LRRFS, HR = 1.49 and DMFS, HR = 0.95) but significantly different from the N1 category (before adjusted: OS, HR = 2.65, LRRFS, HR = 2.21 and DMFS, HR = 2.00; after adjusted: OS, HR = 2.76, LRRFS, HR = 2.28 and DMFS, HR = 1.93; Figs. 4A–4C′).

### Survival analyses of overall stages according to the TNM 6th edition and TNM 7th edition

**Figure 5:**
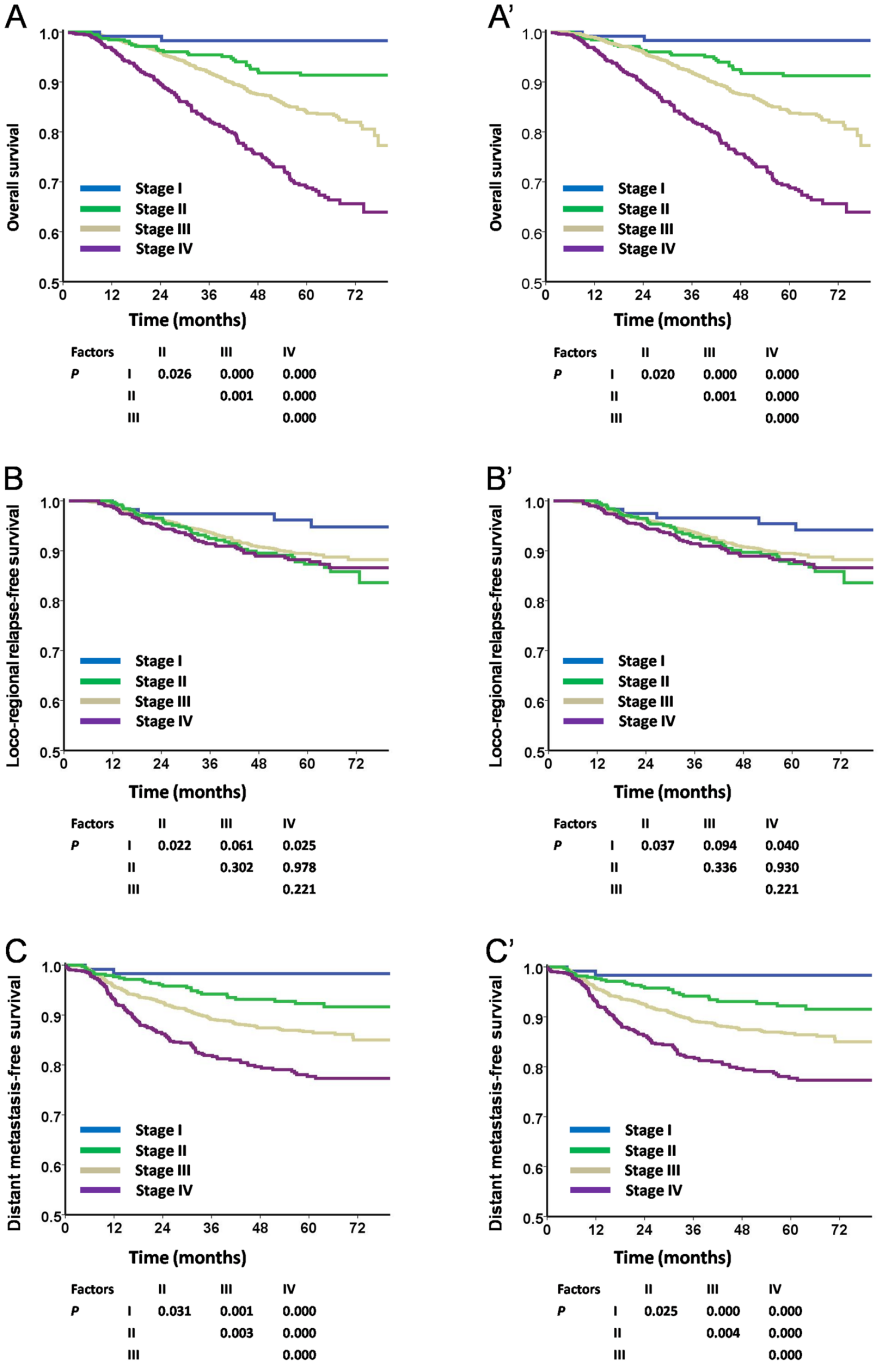
Survival analyses of overall stage using the TNM 6th and the TNM 7th staging systems. **Panels** A, B, and C were staged with the TNM 6th edition staging system, while panels A′, B′, and C′ followed the TNM 7th edition. Both 6th and 7th editions could distinctly separate the survival curves of each overall stage in OS and DMFS (**Panels** 5A and 5A′, 5C and 5C′). With regard to LRRFS, no significant differences were noted among each overall stage except that stage I showed significantly better survival than stage II and stage IV (**Panels** 5B and 5B′).

For OS and DMFS, significant separations were achieved between adjacent stage categories with the TNM 6th edition and TNM 7th edition (P<0.05; Figs. 5A and 5A′, 5C and 5C′, respectively). With respect to LRRFS, the differences were only observed between stage I and stage II (P = 0.022, P = 0.037, respectively) as well as stage I and stage IV (P = 0.025, P = 0.040, respectively) with the two editions of staging systems (Figs. 5B and 5B′).

### Multivariate analysis of T, N, and M categories according to the TNM 6th edition and TNM 7th edition

We conducted a Cox model multivariate analysis after adjusting for gender, age, and treatments and the results revealed that T, N, and M classifications were independent prognostic factors for OS and DMFS by both the TNM 6th edition and TNM 7th edition (P<0.05), but not for LRRFS (P>0.05; Table 3 and S3 Table).

**Table 3:** Multivariate analysis of independent prognostic factors in NPC patients.

		TNM6th				TNM7th			
End point	Variable	Univariate analysis		Multivariate analysis		Univariate analysis		Multivariate analysis	
		HR(95% CI)	*P* value	HR(95% CI)	*P* value	HR(95% CI)	*P* value	HR(95% CI)	*P* value
**Distant failure**	T Classification		<0.001		0.002		<0.001		0.002
	T1	reference		reference		reference		reference	
	T2	2.157(1.225–3.797)	0.008	1.905(1.079–3.366)	0.026	2.099(1.236–3.566)	0.006	1.840(1.080–3.136)	0.025
	T3	2.839(1.675–4.813)	<0.001	2.280(1.333–3.901)	0.003	2.693(1.657–4.377)	<0.001	2.148(1.310–3.521)	0.002
	T4	3.526(2.037–6.103)	<0.001	2.822(1.611–4.943)	<0.001	3.344(2.012–5.559)	<0.001	2.634(1.565–4.431)	<0.001
	N Classification		<0.001		<0.001		<0.001		<0.001
	N0	0.464(0.318–0.677)	<0.001	0.502(0.342–0.736)	<0.001	0.491(0.329–0.734)	0.001	4.679(2.947–7.431)	<0.001
	N1	reference		reference		reference		reference	
	N2	1.305(1.004–1.698)	0.047	1.275(0.978–1.663)	0.073	1.404(1.086–1.814)	0.009	1.358(1.047–1.760)	0.021
	N3	2.741(1.929–3.896)	<0.001	2.378(1.661–3.405)	<0.001	2.948(2.885–4.169)	<0.001	2.530(1.774–3.607)	<0.001
	M Classification								
	M0	reference		reference		reference		reference	
	M1	4.611(3.105–6.846)	<0.001	3.343(2.228–5.015)	<0.001	4.611(3.105–6.846)	<0.001	3.421(2.281–5.132)	<0.001
**Death**	T Classification		<0.001		<0.001		<0.001		<0.001
	T1	reference		reference		reference		reference	
	T2	1.656(0.986–2.780)	0.056	1.641(0.974–2.764)	0.063	1.797(1.088–2.967)	0.022	1.737(1.048–2.877)	0.032
	T3	2.595(1.620–4.156)	<0.001	2.459(1.520–3.977)	<0.001	2.687(1.713–4.216)	<0.001	2.489(1.572–3.942)	<0.001
	T4	4.135(2.549–6.706)	<0.001	4.017(2.442–6.608)	<0.001	4.283(2.695–6.806)	<0.001	4.038(2.503–6.513)	<0.001
	N Classification		<0.001		<0.001		<0.001		<0.001
	N0	0.497(0.357–0.691)	<0.001	0.488(0.349–0.682)	<0.001	0.563(0.400–0.792)	0.001	0.563(0.398–0.796)	0.001
	N1	reference		reference		reference		reference	
	N2	1.200(0.944–1.526)	0.136	1.246(0.977–1.589)	0.076	1.307(1.032–1.655)	0.026	1.359(1.070–1.727)	0.012
	N3	2.761(1.986–3.836)	<0.001	2.478(1.766–3.478)	<0.001	3.007(2.170–4.166)	<0.001	2.696(1.925–3.776)	<0.001
	M Classification								
	M0	reference		reference		reference		reference	
	M1	5.098(3.524–7.374)	<0.001	3.576(2.443–5.234)	<0.001	5.098(3.524–7.374)	<0.001	3.653(2.496–5.345)	<0.001
**Loco-regional relapse**	T Classification		0.114		0.135		0.071		0.079
	T1	reference		reference		reference		reference	
	T2	2.053(1.138–3.705)	0.017	2.026(1.118–3.674)	0.02	2.110(1.202–3.706)	0.009	2.100(1.189–3.707)	0.011
	T3	1.718(0.980–3.011)	0.059	1.747(0.982–3.109)	0.058	1.702(1.002–2.891)	0.049	1.736(1.006–2.998)	0.048
	T4	1.590(0.855–2.955)	0.143	1.629(0.857–3.098)	0.137	1.575(0.871–2.846)	0.133	1.604(0.866–2.972)	0.133
	N Classification		0.035		0.056		0.087		0.169
	N0	0.691(0.459–1.040)	0.077	0.673(0.442–1.023)	0.064	0.831(0.549–1.258)	0.381	0.823(0.538–1.260)	0.37
	N1	reference		reference		reference		reference	
	N2	1.043(0.744–1.463)	0.807	1.035(0.735–1.456)	0.845	1.146(0.821–1.599)	0.423	1.129(0.806–1.582)	0.481
	N3	1.547(0.898–2.664)	0.116	1.479(0.849–2.578)	0.167	1.699(0.990–2.917)	0.054	1.606(0.924–2.792)	0.093
	M Classification								
	M0	reference		reference		reference		reference	
	M1	2.084(0.978–4.441)	0.057	1.695(0.783–3.667)	0.18	2.084(0.978–4.441)	0.057	1.714(0.793–3.706)	0.171

Abbreviation: HR = Hazard Ratio, derived from COX proportional hazard model.

## Discussion

Recently, several studies have compared the TNM 6th edition with the TNM 7th edition. Unfortunately, the results have been heterogeneous. In a panel of 903 Chinese NPC patients, Sun et al. observed non-significant differences in prognosis prediction between T2a and T1 categories using the two editions of the UICC/AJCC staging system [12]. Another recent study by OuYang et al. assessed the prognostic value of the two editions of the TNM staging system by combining analyses of the Akaike information criterion (AIC) and Harrell's concordance index (c-index). They concluded that the TNM 6th edition T-classification had superior prognostic value to the TNM 7th edition. However, FFS was the only factor evaluated following end points [22]. Since clearly elucidating the superiority of the current staging system is critical for treatment choice and prognosis prediction [23], [24], we performed a retrospective study with a large sample size of 2,629 NPC patients. In this study, we compared the prognostic value of the TNM 6th edition and TNM 7th edition based on the significant separations of survival curves for OS, LRRFS and DMFS, which were the most widely used practices in cancer research. Our study revealed that there was a significant difference and better survival curve segregations between T1 and T2 (for OS), N1and N2 (for OS and DMFS) according to the TNM 7th edition.

Considering suggestions from previous studies regarding the prognosis of certain features in TNM 6th edition staging criteria [25], [26], [27], it was not unexpected to observe improvement with the TNM 7th edition, and our aim was to validate these results.

For the T-classification, our study revealed that with the TNM 7th edition, a significant difference and better survival curve segregation between T1 and T2 were obtained in OS analysis. Moreover, the rationality of the TNM 7th edition was reconfirmed based on the findings of the proportional hazards analyses. These observations were consistent with a series of previous studies, where they found no significant differences between T2a and T1 categories in the risks of disease failures and survival rates, and proposed that T2a should be categorized as T1 [25], [26], [27], [28], [29]. On the contrary, Sun et al. did not find significant differences between T2a and T1 in either of the two editions. The discordance might be partly due to the different sample sizes, where a total of 2,629 patients were recruited in our study but only 903 patients participated in their study. Interestingly, reverse results were obtained by OuYang et al. As different following end points and statistical models were used between the two studies, further research using the data of patients from other centers is required.

With regard to N-classification, subsequent retrospective studies revealed that RLN metastasis, regardless of laterality, had a poorer prognosis than node-negative disease [30], [31], [32], [33]. However, reverse evidence showed that RLN metastases did not influence OS and DMFS [12], [34], [35]. Because of the uncertain prognostic value of RLN metastasis, we classified patients with positive RLN as a subgroup alone, and found the similarity of risks of disease failure between N0_RLN+_ and N0_RLN-_ , but different from N1. In addition, our results also revealed that classifying RLN metastasis to N1 obtained better survival curve separations between N1 and N2 in OS and DMFS according to the TNM 7th edition. The following reasons may explain the heterogeneous results regarding the prognostic value of RLN metastasis between our model and previous studies. On the one hand, cumulative studies have reported that the use of MRI was associated with improved tumor control and survival of NPC patients because a higher radiation dose could be delivered to the tumor by the external beam, which led to better tumor control [36], [37]. On the other hand, the visible RLNs were outlined with the primary tumor as GTV and received higher dose irradiation than cervical lymph nodes using either IMRT or 2D-CRT techniques, which eventually led to satisfactory control of RLN. Therefore, it is acceptable that RLN involvement did not significantly influence the prognosis of NPC patients due to the improvement of tumor extent visualization. Further validations with data from multiple centers are required and we are currently planning these studies.

With respect to overall stage, both editions had distinct survival curves in OS and DMFS, which is consistent with the previous findings by Rui et al. [38]. However, with regard to LRRFS, neither edition had significant survival curves separations. An important reason might be the increased local control rates with benefits from the improvement of imaging and radiotherapy modalities and the widespread use of combined chemo-radiotherapy. It has been suggested that MRI had an influence on local tumor control, with around a 20% improvement in the local control rate for NPC [39]. 3D-CRT and IMRT had remarkable advantages in providing better tumor target coverage and allowed the delivery of a high dose to the gross tumor while significantly reducing the dose to the surrounding normal tissues [40], [41]. Therefore, it is urgent to search for a marker to improve the prognostic predication of LRRFS, which is what we are currently planning.

## Conclusion

The TNM 7th staging system had superior performance to the TNM 6th edition in predicting OS and DMFS, and significant alterations in the distribution of N classification. As these findings were limited due to being a retrospective study in a single center, further studies in multicenters or employing a prospective method are needed to validate these findings.

## Supporting Information

S1 Table Baseline clinical characteristics of the 2,671 nasopharyngeal carcinoma patients.
(DOC)

S2 Table Comparison of the survival rate by TNM 6th and TNM 7th staging system.
(DOC)

S3 Table Multivariate analysis of independent prognostic factors in NPC patients.
(DOC)
