# Supplementary material for: A Comparison between the Sixth and Seventh Editions of the UICC/AJCC Staging System for Nasopharyngeal Carcinoma in a Chinese Cohort
Source: PLoS One. 2014 Dec 23;9(12):e116261. doi: 10.1371/journal.pone.0116261 (PMC4275293; doi:10.1371/journal.pone.0116261)
Supplement: S1 Table — Baseline clinical characteristics of the 2,671 nasopharyngeal carcinoma patients. (DOC) [file pone.0116261.s001.doc]

| **S1 Table. Baseline clinical characteristics of the 2,671**  **nasopharyngeal carcinoma patients** | | | | | | |
| --- | --- | --- | --- | --- | --- | --- |
| **Characteristic** | TNM 6th edition | | TNM 7th edition | |  |  |
|  | N | % | N | % | χ^2^ | P |
| **Gender** |  |  |  |  |  |  |
| Female | 685 | 34.50 | 685 | 34.50 |  |  |
| Male | 1,986 | 65.50 | 1,986 | 65.50 |  |  |
| **Age (year)** |  |  |  |  |  |  |
| Median | 50 |  | 50 |  |  |  |
| Range | 6-86 |  | 6-86 |  |  |  |
| **Histology** |  |  |  |  |  |  |
| WHO type I | 10 | 0.40 | 10 | 0.40 |  |  |
| WHO type II | 132 | 4.90 | 132 | 4.90 |  |  |
| WHO type III | 2,529 | 94.70 | 2,529 | 94.70 |  |  |
| **T category*** |  |  |  |  | 3.281 | 0.350 |
| T1 | 292 | 11.10 | 329 | 12.50 |  |  |
| T2 | 561 | 21.30 | 524 | 19.90 |  |  |
| T3 | 1,248 | 47.50 | 1,248 | 47.50 |  |  |
| T4 | 530 | 20.10 | 530 | 20.10 |  |  |
| **N category*** |  |  |  |  | 20.589 | <0.001 |
| N0 | 670 | 25.40 | 551 | 20.90 |  |  |
| N1 | 728 | 27.70 | 847 | 32.20 |  |  |
| N2 | 1,044 | 39.70 | 1,044 | 39.70 |  |  |
| N3 | 189 | 7.20 | 189 | 7.20 |  |  |
| **M category*** |  |  |  |  |  |  |
| M0 | 2,554 | 97.10 | 2,554 | 97.10 |  |  |
| M1 | 77 | 2.90 | 77 | 2.90 |  |  |
| **Overall stage*** |  |  |  |  |  |  |
| I | 118 | 4.50 | 120 | 4.60 | 0.641 | 0.887 |
| II | 389 | 14.80 | 387 | 14.70 |  |  |
| III | 1,401 | 53.20 | 1,401 | 53.20 |  |  |
| IV | 723 | 27.50 | 723 | 27.50 |  |  |
| *Some data are missing | | |  |  |  |  |
| Abbreviations: AJCC = American Joint Committee on Cancer; WHO = World Health Organization. | | | | |  |  |
