# Supplementary material for: A Comparison between the Sixth and Seventh Editions of the UICC/AJCC Staging System for Nasopharyngeal Carcinoma in a Chinese Cohort
Source: PLoS One. 2014 Dec 23;9(12):e116261. doi: 10.1371/journal.pone.0116261 (PMC4275293; doi:10.1371/journal.pone.0116261)
Supplement: S2 Table — Comparison of the survival rate by TNM 6th and TNM 7th staging system. (DOC) [file pone.0116261.s002.doc]

| **S2 table. Comparison of the survival rate by TNM 6th and TNM 7th staging system** | | | | | | | |
| --- | --- | --- | --- | --- | --- | --- | --- |
|  | 3-year survival rate (%) | | |  | 5-year survival rate (%) | | |
|  | OS | LRRFS | DMFS |  | OS | LRRFS | DMFS |
| **TNM 6th** |  |  |  |  |  |  |  |
| T classification |  |  |  |  |  |  |  |
| T1 | 93.2 | 96.5 | 94.9 |  | 93.2 | 93.2 | 94.4 |
| T2 | 88.9 | 90.5 | 89.1 |  | 86.7 | 87.2 | 87.4 |
| T3 | 85.0 | 91.4 | 85.6 |  | 79.4 | 89.1 | 83.2 |
| T4 | 79.2 | 92.6 | 82.1 |  | 67.3 | 91.0 | 80.6 |
| N classification |  |  |  |  |  |  |  |
| N0 | 93.8 | 94.2 | 94.5 |  | 88.8 | 92.0 | 92.9 |
| N1 | 86.2 | 92.1 | 87.1 |  | 79.8 | 89.0 | 84.7 |
| N2 | 83.2 | 91.1 | 84.0 |  | 78.4 | 88.8 | 82.9 |
| N3 | 63.9 | 88.5 | 71.0 |  | 55.2 | 84.9 | 63.8 |
| **TNM 7th** |  |  |  |  |  |  |  |
| T classification |  |  |  |  |  |  |  |
| T1 | 93.3 | 96.2 | 94.8 |  | 93.3 | 93.3 | 93.9 |
| T2 | 88.5 | 90.2 | 88.7 |  | 86.0 | 86.6 | 87.2 |
| T3 | 85.0 | 91.4 | 85.6 |  | 79.4 | 89.1 | 83.2 |
| T4 | 79.2 | 92.6 | 82.1 |  | 67.3 | 91.0 | 80.6 |
| N classification |  |  |  |  |  |  |  |
| N0 | 93.3 | 93.7 | 94.6 |  | 88.6 | 91.2 | 93.1 |
| N1 | 87.4 | 92.7 | 87.9 |  | 81.1 | 89.9 | 85.5 |
| N2 | 83.2 | 91.1 | 84.0 |  | 78.4 | 88.8 | 82.9 |
| N3 | 63.9 | 88.5 | 71.0 |  | 55.2 | 84.9 | 63.8 |
| Abbreviation: OS=Overall Survival, LRRFS=Local Regional Recurrence-free survival, DMFS=Distant Metastasis-free survival | | | | | | | |
