# Supplementary material for: A Comparison between the Sixth and Seventh Editions of the UICC/AJCC Staging System for Nasopharyngeal Carcinoma in a Chinese Cohort
Source: PLoS One. 2014 Dec 23;9(12):e116261. doi: 10.1371/journal.pone.0116261 (PMC4275293; doi:10.1371/journal.pone.0116261)
Supplement: S3 Table — Multivariate analysis of independent prognostic factors in NPC patients. (DOC) [file pone.0116261.s003.doc]

| **S3 Table. Multivariate analysis of independent prognostic factors in NPC patients** | | | | | | | | | |
| --- | --- | --- | --- | --- | --- | --- | --- | --- | --- |
|  |  | **TNM6th** | | | | **TNM7th** | | | |
| **End point** | **Variable** | **Univariate analysis** | | **Multivariate analysis** | | **Univariate analysis** | | **Multivariate analysis** | |
|  |  | **HR(95% CI)** | ***P* value** | **HR(95% CI)** | ***P* value** | **HR(95% CI)** | ***P* value** | **HR(95% CI)** | ***P* value** |
| **Distant failure** | Gender (female vs. male) | 1.568(1.191-2.063) | 0.001 | 1.441(1.093-1.901) | 0.01 | 1.568(1.191-2.063) | 0.001 | 1.447(1.097-1.909) | 0.009 |
|  | Age (years)(<51 vs. ≥51) | 1.038(0.837-1.288) | 0.732 | 1.076(0.866-1.337) | 0.507 | 1.038(0.837-1.288) | 0.732 | 1.077(0.867-1.338) | 0.504 |
|  | Histology (DC vs. UDC) | 0.735(0.481-1.123) | 0.155 | 0.723(0.473-1.105) | 0.134 | 0.735(0.481-1.123) | 0.155 | 0.721(0.472-1.103) | 0.132 |
|  | Treatment modality (RT vs. CRT) | 1.802(0.808-1.485) | ＜0.001 | 1.081(0.795-1.469) | 0.619 | 1.802(1.354-2.397) | ＜0.001 | 1.101(0.810-1.496) | 0.541 |
|  | Radiotherapy techniques (CRT vs. IMRT) | 1.037(0.796-1.350) | 0.789 | 1.101(0.842-1.440) | 0.482 | 1.037(0.796-1.350) | 0.789 | 1.112(0.850-1.454) | 0.439 |
| **Death** | Gender (female vs. male) | 1.943(1.487-2.540) | ＜0.001 | 1.764(1.346-2.311) | ＜0.001 | 1.943(1.487-2.540) | ＜0.001 | 1.778(1.356-2.329) | ＜0.001 |
|  | Age (years)(<51 vs. ≥51) | 1.808(1.471-2.221) | ＜0.001 | 1.811(1.471-2.230) | ＜0.001 | 1.808(1.471-2.221) | ＜0.001 | 1.817(1.476-2.236) | ＜0.001 |
|  | Histology (DC vs. UDC) | 0.767(0.515-1.141) | 0.19 | 0.748(0.502-1.114) | 0.153 | 0.767(0.515-1.141) | 0.19 | 0.749(0.503-1.116) | 0.155 |
|  | Treatment modality (RT vs. CRT) | 1.341(1.059-1.699) | 0.015 | 0.727(0.562-0.940) | 0.015 | 1.341(1.059-1.699) | 0.015 | 0.741(0.573-0.960) | 0.023 |
|  | Radiotherapy techniques (CRT vs. IMRT) | 0.812(0.620-1.064) | 0.131 | 0.935(0.711-1.229) | 0.631 | 0.812(0.620-1.064) | 0.131 | 0.945(0.719-1.243) | 0.688 |
| **Loco-regional relapse** | Gender (female vs. male) | 1.734(1.203-2.500) | 0.003 | 1.764(1.211-2.549) | 0.003 | 1.734(1.203-2.500) | 0.003 | 1.775(1.228-2.565) | 0.002 |
|  | Age (years)(<51 vs. ≥51) | 0.943(0.712-1.248) | 0.681 | 0.927(0.699-1.229) | 0.598 | 1.808(1.471-2.221) | ＜0.001 | 0.921(0.694-1.221) | 0.566 |
|  | Histology (DC vs. UDC) | 0.689(0.400-.186) | 0.179 | 0.680(0.394-1.173) | 0.166 | 0.767(0.515-1.141) | 0.19 | 0.680(0.394-1.173) | 0.166 |
|  | Treatment modality (RT vs. CRT) | 0.976(0.714-1.333) | 0.879 | 0.783(0.552-1.112) | 0.171 | 0.976(0.714-1.333) | 0.879 | 0.81(0.57-1.151) | 0.240 |
|  | Radiotherapy techniques (CRT vs IMRT) | 0.685(0.459-1.022) | 0.064 | 0.704(0.469-1.056) | 0.089 | 0.685(0.459-1.022) | 0.064 | 0.703(0.469-1.055) | 0.089 |
| Abbreviation: DC=differentiated carcinoma; UDC=undifferentiated carcinoma; CRT=chemoradiotherapy; RT= radiotherapy; | | | | | | | | | |
| HR=Hazard Ratio, derived from COX proportional hazard model. | | | | | | | | | |
